# Climate variability, pastoral and agro-pastoral livestock systems, and human nutrition in African drylands: a PRISMA-based systematic review of evidence, pathways, and policy implications

**DOI:** 10.3389/fnut.2026.1853901

**Published:** 2026-06-16

**Authors:** Daniel O. Omokpariola

**Affiliations:** Department of Production and Technical, Office Chérifien des Phosphates (OCP) Africa, Abuja, Nigeria

**Keywords:** African drylands, animal-source foods, climate variability, livestock-mediated nutrition, One Health, pastoralism

## Abstract

Livestock-based livelihoods underpin food security, dietary quality, and resilience in African arid and semi-arid lands (ASALs), yet the mechanisms linking livestock systems, food environments, and human nutrition under climate variability remain fragmented. This PRISMA-based systematic review synthesizes evidence on the nutritional ecology of pastoralist and agro-pastoralist communities in Sub-Saharan Africa, focusing on livestock-mediated nutrition pathways shaped by ecological and socioeconomic change. Literature published between 2006 and 2026 was systematically identified from PubMed, Scopus, AJOL, CABI, and Google Scholar, yielding 60 studies for qualitative synthesis. Nutrition outcomes assessed across the reviewed studies included Household Dietary Diversity Score (HDDS), child wasting and stunting, milk intake, micronutrient adequacy, and food security indicators. The evidence shows that livestock support household nutrition through interconnected biological and economic pathways, including direct consumption of animal-source foods, particularly milk, income generation for food purchases, and buffering against seasonal and climate-related shocks. Larger and more diversified herds are generally associated with higher dietary diversity and improved child nutrition, but benefits are strongly mediated by mobility, market access, gendered control of livestock income, and environmental sustainability. Seasonal dynamics drive marked nutritional variation, with wet seasons supporting improved dietary quality and dry seasons linked to heightened food insecurity and child wasting. Constraints on mobility, sedentarization, market disruptions, and environmental degradation further amplify nutrition risks. The evidence base is dominated by cross-sectional study designs, limiting causal inference and constraining understanding of how livestock ownership, environmental conditions, and nutrition outcomes co-evolve across seasons and shocks. The review identifies key evidence gaps, including limited longitudinal studies, underrepresentation of urbanizing pastoralists, and minimal attention to youth nutrition and livelihood transitions. Drawing on a One Health and food-systems framework, addressing these gaps requires integrated nutrition–livestock surveillance and multisectoral policies to advance climate-resilient, nutrition-sensitive livestock development and support progress toward Sustainable Development Goals related to zero hunger, good health and well-being, and climate action in African drylands.

## Graphical abstract

**Figure:**
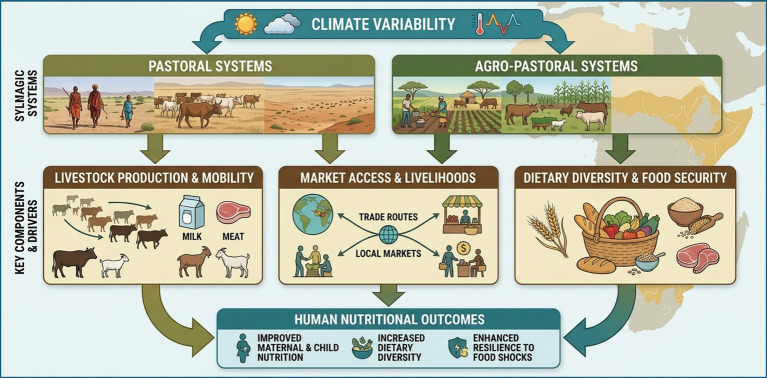

## 1 Introduction

### 1.1 Nutritional ecology in African arid and semi-arid lands

Nutritional ecology provides a framework for understanding how ecological conditions, resource availability, and livelihood strategies jointly shape dietary intake, nutritional status, and health outcomes in human populations (1). Originally rooted in ecological and evolutionary sciences, nutritional ecology emphasizes interactions between organisms and their food environments, including the quantity, quality, and seasonal variability of nutrients, as well as the adaptive strategies used to cope with resource uncertainty (2). In human populations dependent on climatically sensitive ecosystems, nutritional ecology offers a systems-based lens for linking environmental dynamics, food systems, and nutrition outcomes across spatial and temporal scales (3).

African arid and semi-arid lands (ASALs) cover approximately 43–45% of the continent and support over 300 million people, many of whom depend directly on livestock-based livelihoods for food, income, and cultural identity (4, 5). These environments are characterized by low and highly variable rainfall, recurrent droughts, fragile rangelands, and strong seasonal fluctuations in biomass and water availability (6). Such conditions expose populations to recurrent nutrition stress through disruptions to food availability, access, and utilization, making ASALs critical contexts for applying nutritional ecology perspectives (7).

Livestock play a significant role in the nutritional ecology of ASALs by acting as biological converters of sparse and spatially heterogeneous forage into nutrient-dense animal-source foods such as milk, meat, and blood (8). These foods contribute high-quality protein and essential micronutrients, including iron, zinc, vitamin A, and vitamin B_12_, which are critical for child growth, cognitive development, and maternal health (9). At the same time, livestock serve as financial assets that buffer households against food insecurity by enabling food purchases during periods of crop failure or seasonal scarcity (10). Consequently, disruptions to ecological conditions, particularly those driven by climate variability, can rapidly cascade through livestock systems to reshape diets and nutrition outcomes via interconnected biological, economic, and environmental pathways, underscoring the relevance of nutrition-sensitive and One Health-informed livestock systems thinking (11).

### 1.2 Pastoralist and agro-pastoralist livelihood systems in Africa

Pastoralist and agro-pastoralist systems represent the dominant livelihood strategies in African drylands and reflect long-standing adaptations to climatic uncertainty and ecological variability (12). Pastoralism is defined by extensive livestock production systems that rely primarily on natural rangelands and strategic mobility to access seasonally available pasture and water (13). Agro-pastoralism, by contrast, integrates livestock keeping with limited crop cultivation, often as a risk-management strategy in transitional zones between arid and sub-humid areas (14). Pastoral systems are particularly adapted to high climate variability because mobility enables herders to track spatially and temporally uneven resource distributions, thereby minimizing livestock losses and stabilizing food production over time (15). Where mobility is maintained, pastoral households can better sustain livestock productivity and seasonal food availability despite climatic shocks. In contrast, agro-pastoral households often experience greater exposure to climatic shocks due to partial sedentarization, land fragmentation, and increasing reliance on rain-fed crops (16). Evidence from East and West Africa shows that transitions from pastoralism to agro-pastoralism can improve income diversification but may also alter livestock-mediated nutrition pathways, reducing dietary diversity and resilience if livestock productivity declines or mobility is constrained (17).

Nutrition outcomes within these systems are heterogeneous and shaped by multiple interacting pathways, including herd composition, market access, gendered control of livestock products, and seasonal labour demands (18). Pastoralist households with greater access to milk-producing animals often exhibit higher dietary diversity and improved child nutrition status during normal seasons, but these benefits can erode rapidly during prolonged droughts or disease outbreaks (19). Agro-pastoralists may benefit from crop-based calories yet often face micronutrient deficiencies when livestock ownership or access to animal-source foods is limited (20). Distinguishing between pastoralist and agro-pastoralist livelihood systems is therefore essential for accurately interpreting livestock–nutrition relationships and for informing context-specific nutrition-sensitive policy and program design.

### 1.3 Livestock–food environment–nutrition linkages under climate variability

The livestock–food environment–nutrition nexus is increasingly recognized as a critical pathway linking ecological processes to human health in African drylands (21). Climate variability, including drought, heat stress, and erratic rainfall, directly affects rangeland productivity, water availability, and animal health, thereby influencing livestock outputs such as milk yield, growth rates, and mortality (22). Indirect effects include altered disease dynamics, reduced forage quality, and disruptions to livestock markets, all of which shape household food environments (23). Together, these processes illustrate how climate stressors operate through both production and access pathways to influence nutrition outcomes. These pathways have profound nutritional implications. Studies across the Horn of Africa and the Sahel demonstrate that drought-induced declines in milk availability are strongly associated with increased child wasting and reduced dietary diversity (24). While these associations are robust across contexts, most studies are observational, limiting direct causal attribution.

Market-mediated pathways are equally important; livestock sales during climate shocks can provide short-term income for food purchases, but distress sales may undermine long-term livelihood resilience and future food security (25). Gender dimensions further mediate these outcomes, as women’s control over milk income and food allocation often determines whether livestock benefits translate into improved child and maternal nutrition (26). This highlights the importance of intra-household dynamics in shaping nutrition outcomes beyond aggregate livestock ownership or income measures. Despite growing empirical evidence, the literature remains fragmented, with climate variability, livestock production, and nutrition outcomes often analyzed in isolation (27). Existing reviews tend to focus on either climate impacts on livestock systems or nutrition outcomes in isolation, rather than systematically synthesizing how climate variability reshapes nutritional ecology through livestock-mediated food environments (28). This gap limits the ability of policymakers and development practitioners to design nutrition-sensitive, gender-responsive, and climate-resilient livestock interventions.

### 1.4 Relevance to sustainable development goals (SDGs 2, 3, and 13)

Understanding nutritional ecology in pastoralist and agro-pastoralist systems is central to achieving multiple interconnected Sustainable Development Goals. SDG 2 (Zero Hunger) emphasizes not only food availability but also access to nutritious diets, resilience of food systems, and sustainable agricultural practices (29). Livestock systems in ASALs contribute substantially to local and regional food supply, particularly of animal-source foods that are critical for reducing undernutrition and micronutrient deficiencies (30), directly aligning with SDG target 2.1 (ending hunger) and 2.2 (ending all forms of malnutrition). SDG 3 (Good Health and Well-Being) is intricately linked to dietary quality and early-life nutrition. Undernutrition remains a major driver of child morbidity and mortality in dryland regions, where climate shocks frequently reverse nutrition gains (31). Strengthening livestock-based nutrition pathways while minimizing food safety risks offers a powerful lever for improving health outcomes in these populations (32), particularly in relation to SDG target 3.2 on preventable child mortality. SDG 13 (Climate Action) highlights the need for adaptation strategies that protect vulnerable livelihoods while maintaining ecosystem sustainability (33). Pastoral and agro-pastoral systems are both highly exposed and inherently adaptive to climate variability, yet policy interventions often fail to leverage their adaptive capacities (34). Integrating nutritional outcomes into climate-livestock policies can support co-benefits for food security, health, and climate resilience (35), contributing to SDG target 13.1 on strengthening resilience and adaptive capacity to climate-related hazards.

### 1.5 Aim and objectives of the review

Against this backdrop, this study aims to systematically synthesize evidence on the nutritional ecology of African pastoralist and agro-pastoralist systems under climate variability, with a specific focus on livestock–food environment–nutrition linkages across arid and semi-arid lands. Using a PRISMA-guided systematic review approach, the review seeks to address the following objectives:

1. To examine how climate variability influences livestock productivity, food environments, and human nutrition outcomes in African ASALs.
2. To compare nutritional pathways and outcomes between pastoralist and agro-pastoralist livelihood systems.
3. To identify key mediating factors such as seasonality, markets, and gender dynamics that shape livestock-based nutrition outcomes.
4. To assess implications for achieving SDGs 2, 3, and 13 through nutrition-sensitive and climate-resilient livestock policies.
5. To highlight evidence gaps and priority areas for future research and integrated policy action.

By explicitly integrating climate variability, livestock systems, and nutrition outcomes within a nutritional ecology and One Health framework, this review provides a structured and policy-relevant lens for understanding how livestock systems can support resilient, healthy, and sustainable food systems in African drylands.

## 2 Methods

### 2.1 Review design and reporting framework

This study was conducted as a systematic review and reported in accordance with the Preferred Reporting Items for Systematic Reviews and Meta-Analyses (PRISMA) 2020 guidelines, which provide a standardized framework for transparent identification, screening, eligibility assessment, and inclusion of studies in evidence syntheses. A formal review protocol was developed prior to literature screening to guide the search strategy, eligibility criteria, and synthesis approach. Although the protocol was not prospectively registered in PROSPERO due to the interdisciplinary scope of the review, all methodological decisions were specified *a priori* and were not modified during the review process. The research question and inclusion criteria were structured using the PICOS framework (population, exposure, comparator, outcomes, and study design), which is summarized in Supplementary materials.

The PRISMA approach was selected because of its widespread application in health, nutrition, and environmental systems research and its suitability for interdisciplinary reviews linking livestock systems, food environments, and human nutrition outcomes in climate-sensitive settings. The overall review design aligns with recent systematic reviews of livestock and climate variability in Africa, including evidence mapping and synthesis approaches applied to pastoral and agro-pastoral systems (36). The review process followed the four PRISMA phases of identification, screening, eligibility, and inclusion. To address transparency and reproducibility, methodological steps and study flow are documented using structured tables and figures presented in this manuscript.

### 2.2 Data sources and search strategy

A comprehensive literature search was performed using five electronic databases selected to capture peer-reviewed literature across nutrition, public health, agriculture, and livestock systems research. These databases included PubMed, Scopus, African Journals Online (AJOL), CABI Abstracts, and Google Scholar. The combination of global biomedical databases and agriculture- and Africa-focused repositories was intended to minimize indexing bias and ensure coverage of regionally published studies that are often under-represented in large biomedical databases. Searches employed combinations of free-text terms and truncations related to pastoralism and agro-pastoralism, livestock systems, human nutrition outcomes such as dietary diversity and stunting, food environments, and Africa. Boolean operators were used to structure the search logic, and syntax was adapted where necessary to suit individual database requirements. In addition to database searching, “other sources” comprised manual screening of reference lists of included articles and relevant reviews, as well as targeted searches of institutional reports published in peer-reviewed outlets indexed within the selected databases. To ensure reproducibility, the full search strategy for at least one database (PubMed), including all search terms, truncations, and Boolean operators, is provided in the Supplementary materials. Supplementary searching of reference lists was undertaken to identify additional relevant studies, consistent with best practice in systematic review methodology (36).

### 2.3 Eligibility criteria

Studies were considered eligible for inclusion if they were published between January 2006 and January 2026, focused on Sub-Saharan Africa, examined pastoralist or agro-pastoralist populations, and reported human nutrition outcomes linked directly or indirectly to livestock systems or food environments. The lower time boundary (2006) was selected to capture the period during which nutrition-sensitive agriculture, climate–livestock interactions, and food-systems approaches became more explicitly integrated into peer-reviewed research, following increased global attention to climate change adaptation and nutrition outcomes in dryland systems. Eligible nutrition outcomes included dietary diversity, child stunting or wasting, micronutrient status, and related indicators of nutrition and food security. Only studies published in English or French were included. Empirical quantitative, qualitative, and mixed-methods study designs were considered appropriate for inclusion, reflecting the interdisciplinary nature of nutritional ecology research in dryland systems. Studies were excluded if they focused exclusively on crop-based production systems, were conducted outside arid or semi-arid contexts, lacked human nutrition outcomes, or did not examine livestock-related exposures. Editorials, commentaries, conference abstracts, and other non-peer-reviewed publications were also excluded.

### 2.4 Study selection and descriptive analysis of search results

**Figure 1:**
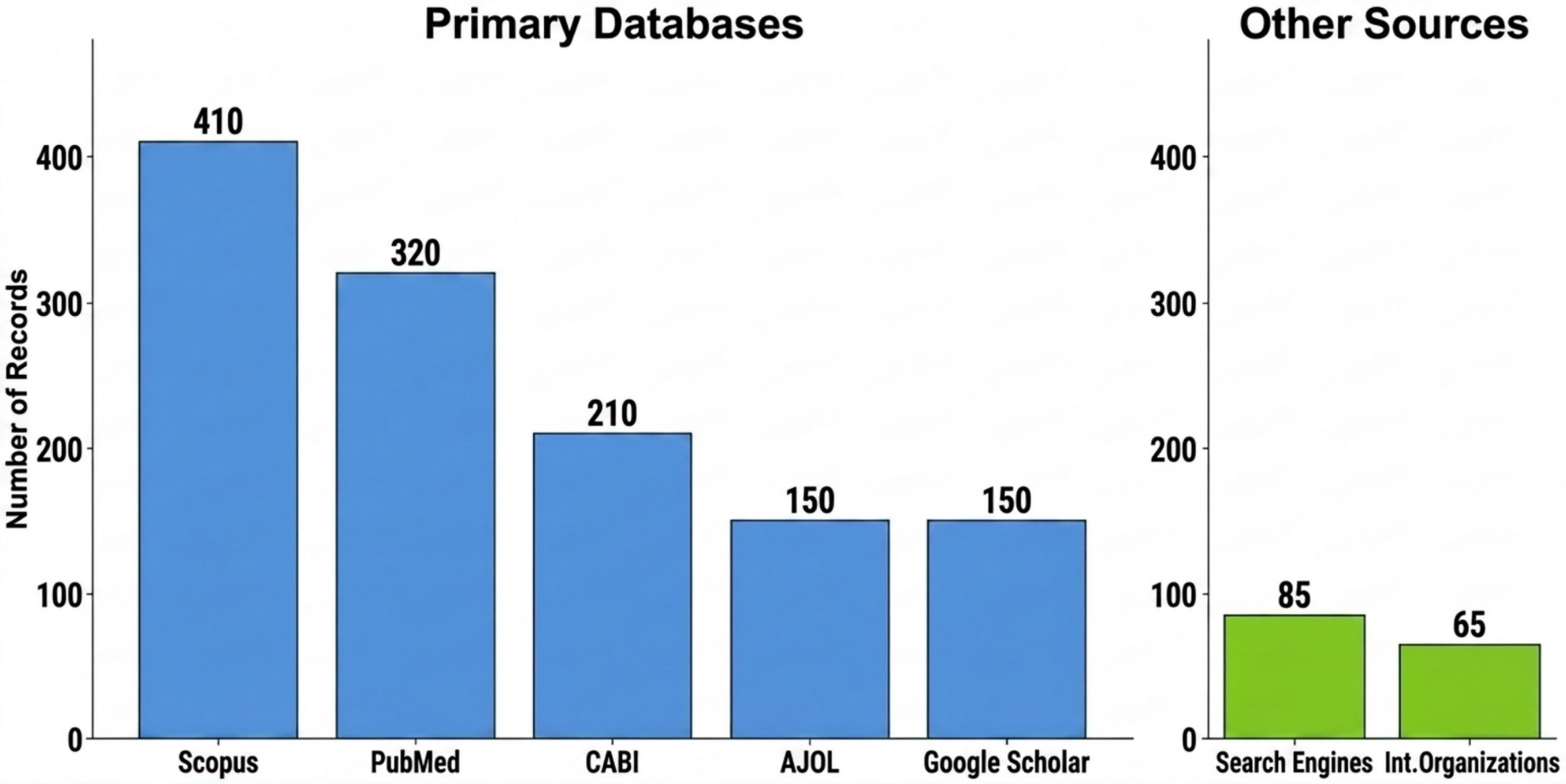
Distribution of records identified across databases.

**Figure 2:**
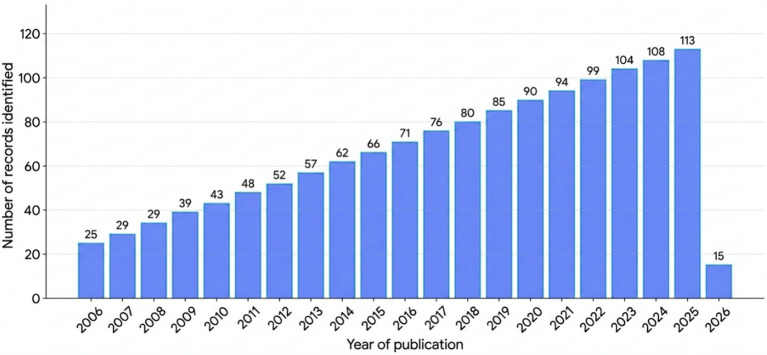
Year-by-year distribution of records identified across all databases.

Following database searching, all retrieved records were collated and duplicates removed before screening. Initial screening was conducted at the title and abstract level to exclude clearly irrelevant studies, followed by full-text assessment of potentially eligible articles using the predefined criteria. Title and abstract screening, full-text eligibility assessment, and data extraction were conducted by the author, with screening decisions cross-checked iteratively against the eligibility criteria to minimize selection bias. Where uncertainty arose regarding study inclusion, eligibility decisions were resolved through re-examination of the study aims, methods, and outcomes in relation to the review objectives. In line with recommendations for enhanced transparency in systematic reviews, descriptive analyses were conducted to characterize the evidence base at the identification stage. The distribution of records identified across databases is presented in Figure 1, illustrating the relative contribution of each data source to the overall search yield. In addition, the temporal distribution of records identified between 2006 and 2026 is shown in Figure 2, highlighting year-by-year trends in the accumulation of searchable literature on pastoralist and agro-pastoralist nutrition and livestock systems. These descriptive figures provide methodological context for the review and mirror approaches used in large Cochrane and climate–livestock evidence syntheses (36).

### 2.5 PRISMA flow diagram and final study inclusion

**Figure 3:**
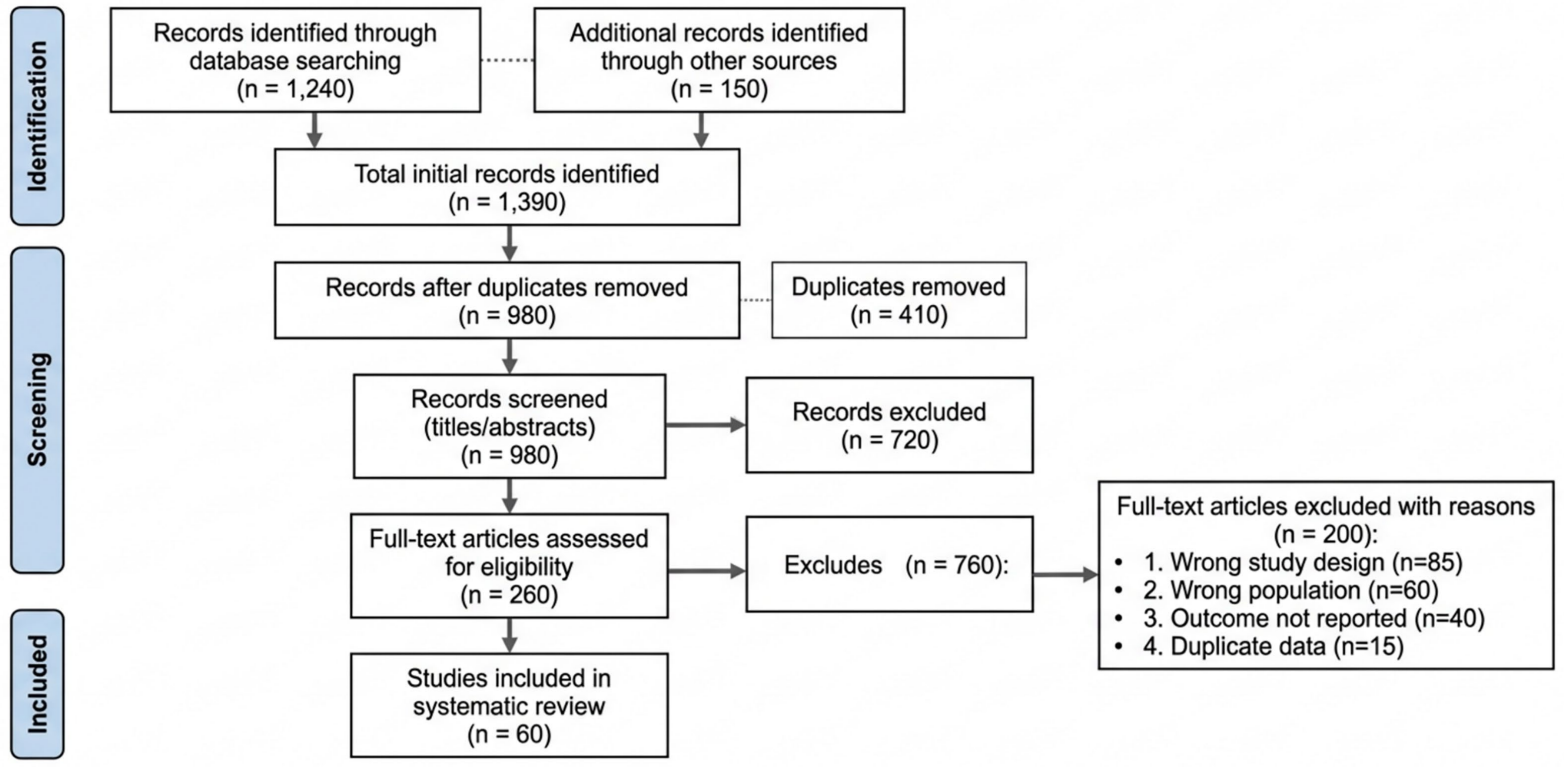
PRISMA flow diagram of study identification, screening, and inclusion.

The progression of studies through the identification, screening, eligibility, and inclusion stages is summarized using a PRISMA flow diagram formatted according to the classic Cochrane style (Figure 3). Electronic database searches yielded 1,240 records, with an additional 150 records identified through supplementary sources. After removal of duplicate records, 980 unique records remained for title and abstract screening, during which 720 records were excluded because they were clearly irrelevant to pastoralist or agro-pastoralist nutrition research. Full-text eligibility assessment was performed for 260 studies, resulting in the exclusion of 200 articles due to reasons including absence of nutrition outcomes, inappropriate geographical or ecological context, or lack of livestock-related exposure variables. Ultimately, 60 studies met all inclusion criteria and were retained for qualitative synthesis in this systematic review. The numerical values and arrows in the PRISMA flow diagram reflect sequential filtering at each review stage rather than parallel exclusion categories, and totals correspond to PRISMA 2020 reporting guidelines. The structure and reporting of this selection process are consistent with PRISMA 2020 guidance and recent systematic reviews examining livestock systems and climate variability in Africa (36). The characteristics of all studies included in the qualitative synthesis, including study design, geographic location, pastoral system type, livestock exposures, nutrition outcomes, and key findings, are summarized in Supplementary Table S2. All counts reported in the PRISMA diagram were cross-checked for internal consistency, with duplicates explicitly quantified and removal steps aligned with PRISMA 2020 reporting structure.

Given the heterogeneity of study designs, contexts, and outcome measures, a formal quantitative risk-of-bias assessment was not conducted. Instead, study quality was appraised narratively, considering methodological transparency, relevance to pastoral and agro-pastoral contexts, and clarity of nutrition outcome measurement. All figures were generated using Microsoft Excel and Power BI, including formatting using Microsoft PowerPoint.

### 2.6 Word cloud

**Figure 4:**
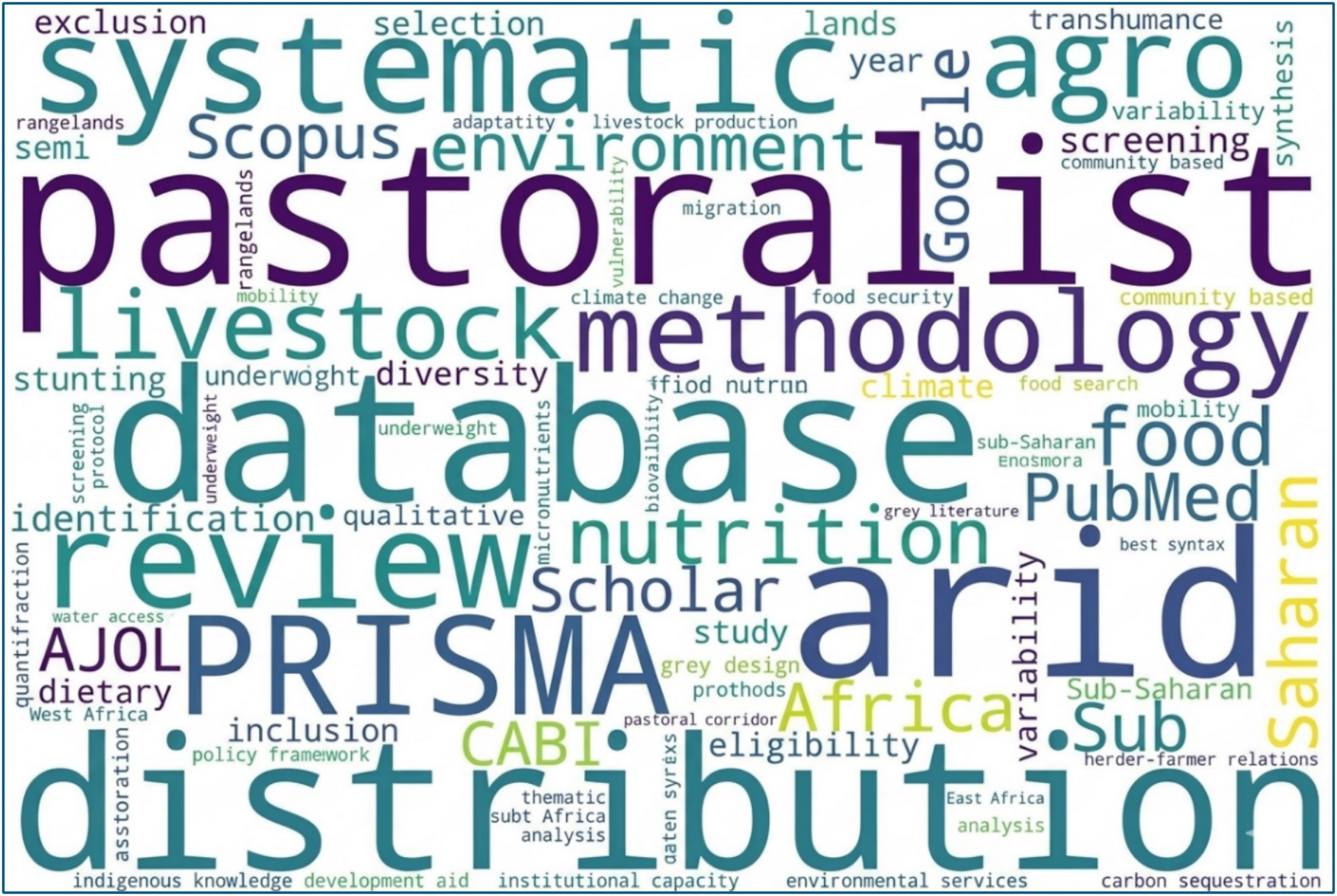
Word cloud of key methodological concepts used in the systematic review.

A word cloud was generated to visually summarize the dominant methodological concepts underpinning the systematic review process (Figure 4). The word cloud was produced using frequency analysis of key terms extracted from titles, abstracts, and methodological descriptors of included studies, serving as a descriptive visualization rather than an analytical or inferential tool. The visualization highlights the prominence of core terms related to PRISMA reporting, pastoralist and agro-pastoralist systems, livestock, nutrition outcomes, food environments, Sub-Saharan Africa, database sources, and study selection stages. This descriptive visualization provides a compact overview of the methodological focus and reinforces the interdisciplinary scope of the review process.

### 2.7 Study design

**Figure 5:**
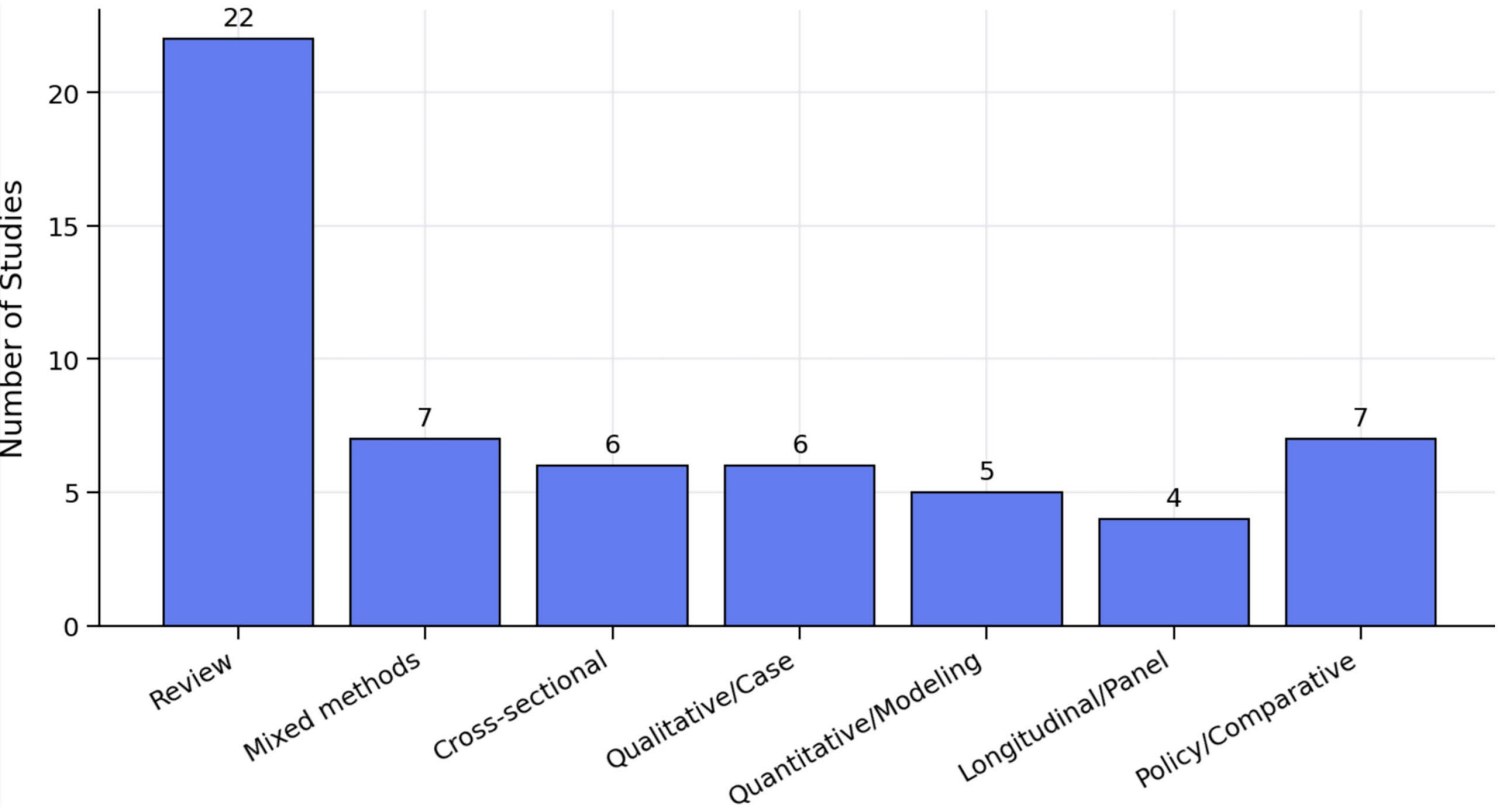
Distribution of study designs across included studies (*n* = 60).

A summary distribution of study designs included in the review is presented in Figure 5, illustrating the predominance of cross-sectional studies relative to longitudinal, mixed-methods, and qualitative designs. This visualization complements the detailed study characteristics provided in Supplementary Table S2 and highlights the methodological imbalance within the evidence base, which has implications for causal inference and evidence interpretation.

## 3 Livestock holdings and household nutrition

### 3.1 Herd size and species composition in relation to household dietary diversity and child nutrition

Across the 60 studies included in this PRISMA-based systematic review, herd size and species composition emerged as frequently reported correlates of household dietary diversity and child nutrition outcomes in pastoralist and agro-pastoralist systems. Larger herd sizes were generally associated with higher Household Dietary Diversity Scores (HDDS) and lower levels of food insecurity, as measured by tools such as the Household Food Insecurity Access Scale (HFIAS), particularly in non-shock years (8, 10, 37, 38). Households owning larger and more diversified herds were more likely to regularly consume animal-source foods and to access a wider range of plant-based foods through market purchases, as reflected in significantly higher dietary diversity scores.

**Figure 6:**
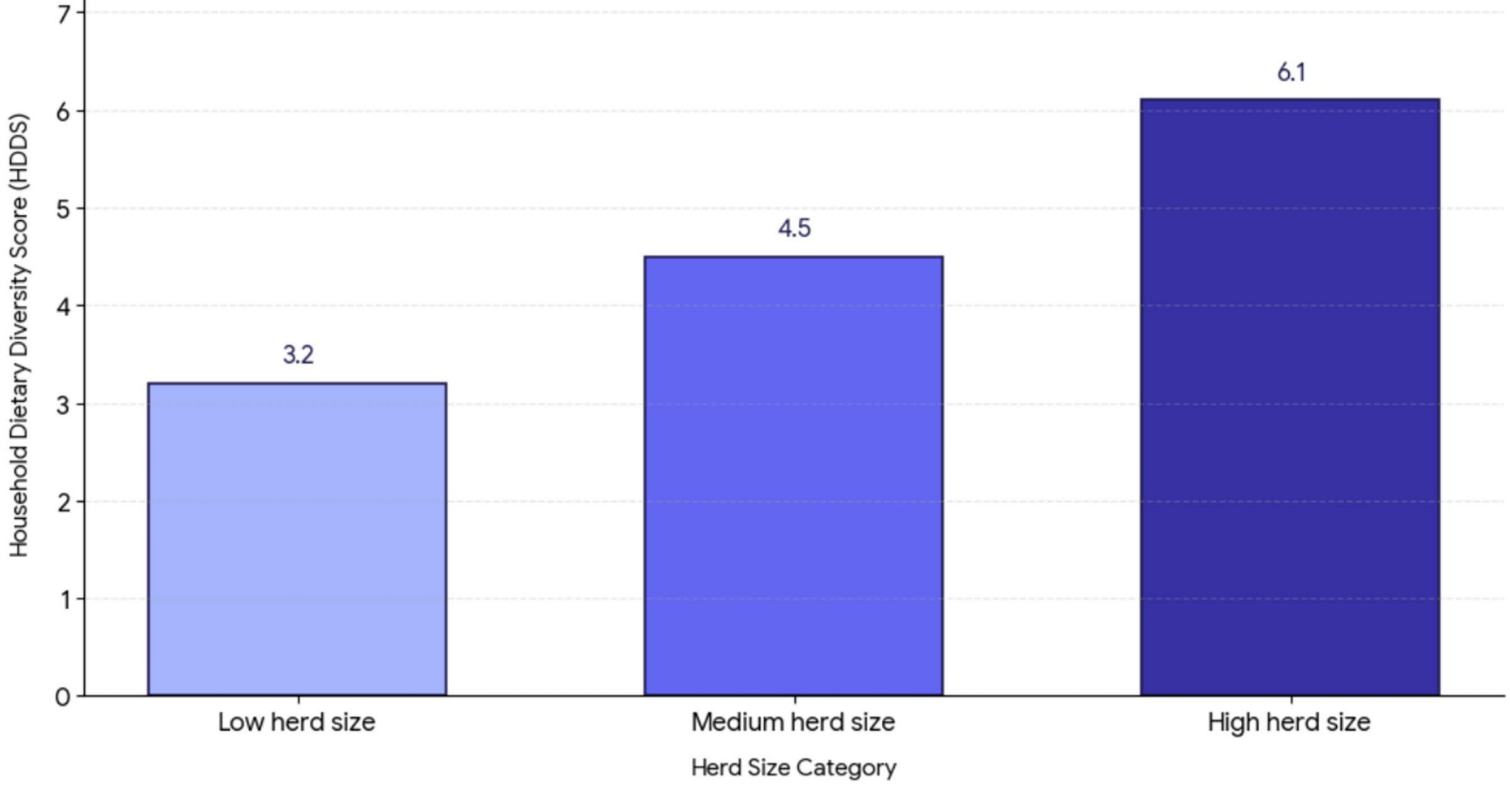
Herd size category and household dietary diversity among pastoralist and agro-pastoralist households. Values represent outcomes from included studies.

Across the reviewed studies, herd size was measured using heterogeneous metrics, including absolute animal counts, Tropical Livestock Units (TLU), and wealth-based livestock indices, with classification thresholds varying by country, ecological zone, and study design. As illustrated in Figure 6, households classified within high herd-size categories consistently achieved dietary diversity scores exceeding six food groups, compared with fewer than four food groups among households with low livestock holdings. These categories reflect relative, study-specific definitions rather than a uniform cross-study threshold.

Species composition further mediated these relationships. Cattle- and camel-dominated systems were most strongly associated with improved dietary diversity due to regular milk availability. In contrast, small-ruminant-dominant systems provided greater liquidity but less consistent access to animal-source foods (31, 39). Randolph et al. (8) reported that households with larger livestock holdings had higher dietary diversity and improved child nutrition outcomes, while Sadler et al. (19) and Nicholson et al. (40) observed lower rates of child wasting and stunting in milk-producing pastoral households, underscoring the role of livestock ownership as a protective factor against chronic undernutrition (9, 37). However, the strength of these associations varied by ecological zone, market access, and household wealth status, suggesting that herd size alone is insufficient to guarantee improved nutrition outcomes in the absence of enabling food environments.

### 3.2 Milk, meat, and blood as direct nutrition pathways

Direct consumption of livestock products constituted a primary biological pathway linking livestock holdings to human nutrition across pastoralist and agro-pastoralist systems. Milk was the most frequently consumed and nutritionally significant animal-source food reported across the reviewed studies, particularly in cattle, camel, and mixed-species systems (41–43). Daily or near-daily milk consumption among children and women was associated with improved intake of high-quality protein, calcium, and key micronutrients, and several studies linked milk availability to reduced odds of child wasting during dry seasons (18, 42). Seasonal declines in milk production were repeatedly associated with sharp deteriorations in child dietary quality, highlighting the sensitivity of nutrition outcomes to livestock productivity and climate variability.

Meat consumption was less frequent and more episodic, often driven by cultural practices, ceremonial slaughter, or distress sales during shocks. Nevertheless, when consumed, meat contributed substantially to protein and iron intake and was occasionally associated with short-term improvements in child weight-for-age indicators (44). In specific pastoral contexts, particularly in parts of the Horn of Africa, the consumption of animal blood was reported as a culturally embedded nutritional practice during periods of food scarcity (45, 46). While evidence on the population-level nutritional contribution of blood consumption remains limited, its repeated documentation across ethnographic and nutrition studies underscores the diversity of livestock-derived nutrition pathways within pastoral nutritional ecology and the importance of culturally specific food practices in shaping diet composition.

### 3.3 Livestock as a financial buffer for food purchase

**Figure 7:**
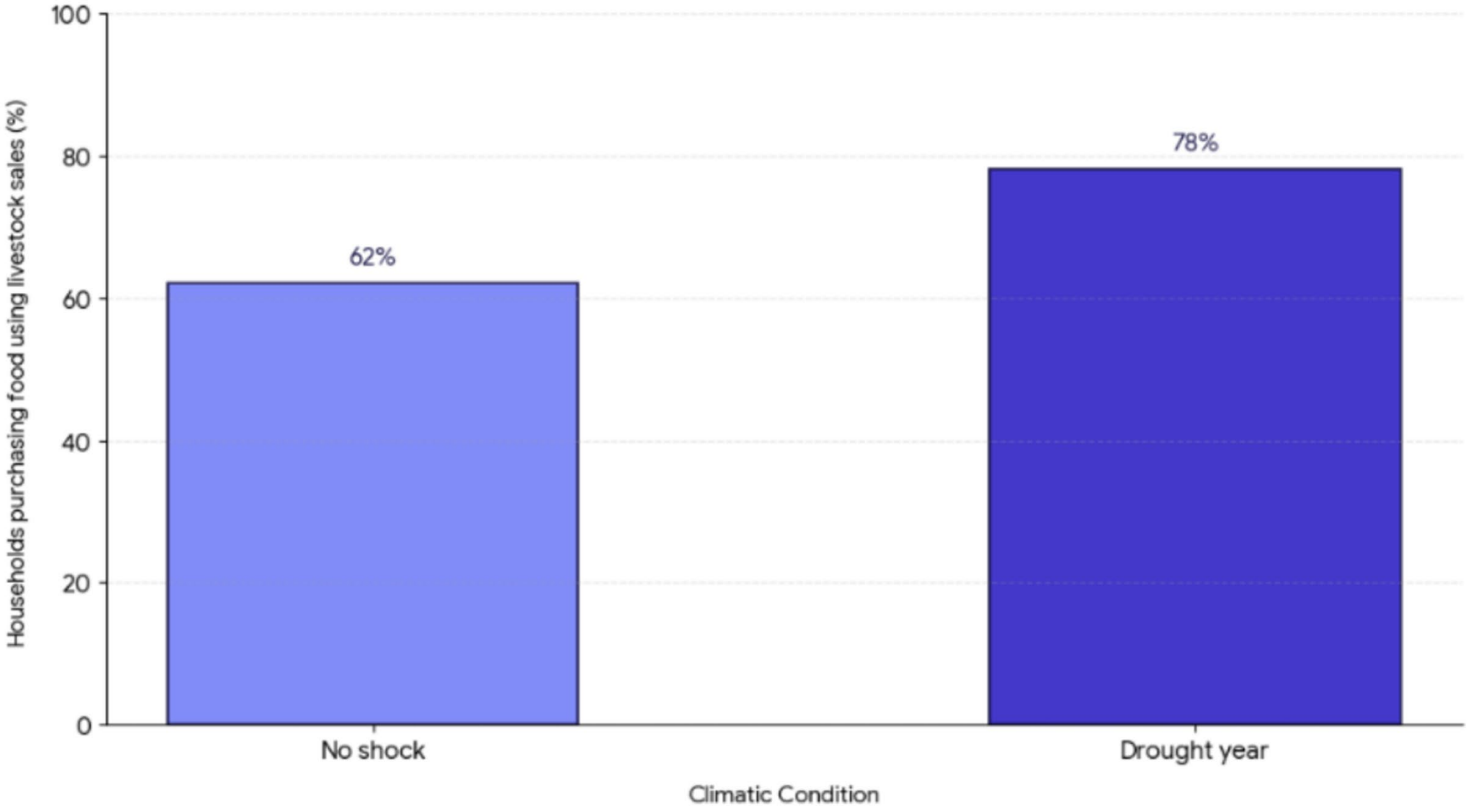
Use of livestock sales as a food security coping mechanism during climate-related shocks.

Beyond direct consumption, livestock holdings played a crucial economic role in stabilizing household food access through their function as a financial buffer. A substantial proportion of included studies documented the strategic sale of livestock or livestock products to purchase cereals, legumes, vegetables, and other non-livestock food items, particularly during droughts, livestock disease outbreaks, and seasonal lean periods (12, 47, 48). This buffering function was especially pronounced among households with diversified herds, as small ruminants could be sold rapidly with relatively limited impact on long-term herd viability (12). Little et al. (25) and McPeak et al. (10) reported that livestock sales were a primary coping strategy for food purchase during drought periods, although repeated distress sales were associated with long-term herd depletion. As shown in Figure 7, the proportion of households relying on livestock sales to finance food purchases increased markedly during climate-related shocks compared with normal seasons, indicating the centrality of livestock as a risk-management asset in dryland food systems.

However, repeated distress sales during prolonged crises were consistently associated with herd depletion, reduced future milk availability, and heightened long-term vulnerability to food insecurity, particularly among poorer households with limited initial livestock assets (23, 24). Gendered control over livestock income further shaped these outcomes, with evidence suggesting that income derived from milk sales often controlled by women, was more strongly associated with improvements in child dietary diversity than income from large animal sales typically managed by men (15, 49). Collectively, these findings emphasize that livestock contribute to household nutrition through intertwined biological and economic pathways, while also exposing households to trade-offs between short-term food access and long-term livelihood and nutrition resilience.

## 4 Mobility, seasonality, and food environments

### 4.1 Transhumance patterns and access to markets and wild foods

Across the studies included in this systematic review, mobility, particularly seasonal transhumance, emerged as a defining feature shaping food environments and nutrition pathways in pastoralist and agro-pastoralist systems. Transhumant movements during the dry season were consistently associated with improved access to grazing resources, sustained livestock productivity, and continued availability of animal-source foods, especially milk (50–52). Niamir-Fuller (15) and Krätli and Schareika (51) reported that seasonal mobility enables pastoral households to track forage availability and sustain milk production during dry periods, a pattern also observed empirically by Little et al. (52). Turner et al. (53) reported that mobility enabled households to maintain milk production during periods when sedentary households experienced sharp declines, thereby buffering seasonal deterioration in dietary quality. In addition to livestock-derived foods, mobile households often accessed wild foods, including edible plants, fruits, and tubers, particularly during migration periods, which contributed modestly but meaningfully to dietary diversity in marginal environments (54). These contributions were highly seasonal and context-specific and typically supplemented rather than replaced livestock-derived nutrition.

Mobility patterns also shaped access to markets in complex ways. While mobile households sometimes faced physical barriers to regular market participation, particularly in remote rangelands, strategic routing of migration often incorporated seasonal proximity to market centers, facilitating livestock and milk sales (55, 56). Studies from East and West Africa documented that households able to synchronize mobility with market access achieved better food purchasing power and more stable food consumption patterns than households whose mobility was restricted by conflict, land fragmentation, or administrative boundaries (48). Conversely, constraints on transhumance disproportionately limited access to both livestock feeds and markets, undermining food environments and increasing reliance on humanitarian assistance (56). These patterns indicate strong associations between mobility, food environments, and nutrition, though pathways vary across ecological and institutional contexts.

### 4.2 Dry versus wet season nutrition outcomes

Seasonality was a dominant driver of variation in nutrition outcomes across pastoralist and agro-pastoralist systems. The wet season was consistently associated with improved dietary diversity and food security, driven by increased pasture availability, higher milk yields, and greater household income from livestock products (57–59). During these periods, households reported more frequent consumption of animal-source foods and greater inclusion of complementary foods such as vegetables and grains, particularly when market access was favorable (60). Correspondingly, several longitudinal studies documented improvements in child weight-for-height indicators during wet seasons, reflecting short-term gains in nutritional status (61). Hoddinott and Kinsey (57) reported seasonal improvements in child growth during wetter periods, while Sellen (58) documented higher dietary intake among pastoral households during seasons of increased pasture and milk availability.

In contrast, the dry season was characterized by reduced dietary diversity, heightened food insecurity, and increased nutritional vulnerability, particularly among children under 5 years of age (62). Declines in milk availability during prolonged dry seasons were repeatedly associated with increased prevalence of wasting and deterioration in child feeding practices (19, 40). Although livestock sales often increased during dry seasons to finance food purchases, these coping strategies were not always sufficient to offset reduced dietary quality, especially when drought conditions coincided with market disruptions or rising food prices (14). Together, these findings highlight seasonality as a structural determinant of nutrition outcomes rather than a temporary or anomalous fluctuation, underscoring the importance of seasonally responsive policy and programmatic interventions.

### 4.3 Effects of sedentarization on diets and nutrition

Sedentarization processes driven by land privatization, settlement programs, security concerns, and expansion of agriculture were widely reported to alter food environments and nutrition outcomes in pastoralist and agro-pastoralist communities (112). Many studies indicated that reduced mobility constrained access to high-quality pastures, leading to declines in livestock productivity and reduced availability of milk and other animal-source foods (63, 64). Sedentarized households were often more reliant on purchased foods, particularly cereals, resulting in diets that were higher in energy but lower in micronutrient density and overall diversity (65).

Evidence on the nutritional implications of sedentarization was mixed but generally suggested increased vulnerability to food insecurity and chronic undernutrition among children (18). While some agro-pastoral households benefited from crop cultivation and improved physical access to markets, these gains were frequently offset by reduced livestock assets and weakened traditional coping mechanisms (14). Several studies also noted that sedentarization disrupted customary systems of resource sharing and mutual support, further constraining food access during shocks (66). Overall, the synthesis indicates that sedentarization reshapes pastoral food environments in context-dependent ways that often reduce dietary diversity and resilience unless accompanied by targeted investments in nutrition-sensitive agriculture, livestock services, mobility-enabling governance arrangements, and market infrastructure, rather than by settlement status alone.

## 5 Market access and gender dimensions

### 5.1 Women’s control over milk income and child nutrition

Across the studies included in this review, women’s control over income derived from milk sales emerged as one of the most consistently reported gendered pathways linking livestock systems to child nutrition outcomes in pastoralist and agro-pastoralist settings. Multiple studies demonstrated that when women retained decision-making authority over milk use and milk income, households exhibited significantly higher dietary diversity scores and improved child feeding practices (26, 66, 67). Njuki et al. (26) reported that women’s control over livestock-derived income, particularly from milk, was associated with improved household food allocation, while Hoddinott et al. (68) documented improved child nutrition outcomes in households where milk income was not mediated through male-dominated markets.

Milk income under women’s control was more likely to be allocated to food purchases, healthcare, and child nutrition needs than income derived from the sale of large livestock, which was typically managed by men (68). This distinction was particularly evident in cattle- and camel-based systems, where regular milk sales provided women with a predictable source of cash income that directly translated into improved dietary quality for children (69). Empirical evidence from East and West Africa consistently showed associations between women’s milk income control and improved child height-for-age and reduced prevalence of stunting, even after adjusting for household wealth and herd size (70, 71).

Conversely, studies documented that erosion of women’s control over milk, often associated with commercialization or shifts toward male-dominated marketing channels, was linked to diminished nutritional gains despite increases in overall household income (72). These findings indicate that commercialization pathways can differentially affect nutrition outcomes depending on intra-household control structures, underscore that livestock commercialization alone does not guarantee improved nutrition outcomes, and that gendered control over livestock-derived resources is a critical mediating factor in the livestock–nutrition pathway.

### 5.2 Distance to markets, livestock traders, and processed food penetration

Market access emerged as a key structural determinant shaping food environments and diet composition in pastoralist and agro-pastoralist communities. Distance to markets and the presence of livestock traders strongly influenced both the capacity of households to sell livestock products and their access to diverse foods (73, 74). Studies consistently reported that households located closer to market centers or seasonal trading hubs exhibited higher dietary diversity, driven by more frequent purchase of cereals, vegetables, legumes, and, increasingly, processed foods (75). Improved market access also facilitated the timely sale of milk and small ruminants, enhancing household liquidity and stabilizing food consumption during seasonal stress (76).

However, growing market integration was also associated with increased penetration of processed and ultra-processed foods, particularly among more sedentary and peri-urban agro-pastoralist populations (77). Several studies noted shifts toward diets higher in refined carbohydrates, sugar, and fats in areas with dense market access, raising concerns about emerging nutrition transitions alongside persistent undernutrition (78). These trends suggest that market integration can simultaneously address caloric deficits while introducing longer-term diet-related health risks. In remote rangelands, limited trader presence constrained livestock commercialization and food purchasing power, reinforcing dependence on own-production of food and humanitarian assistance during shocks (79). Together, these findings highlight market access as a double-edged determinant of nutrition, capable of both enhancing dietary diversity and exacerbating diet-related health vulnerabilities depending on context.

### 5.3 Effects of conflict, drought, and livestock disease on market function

Shocks such as armed conflict, drought, and livestock disease outbreaks were repeatedly identified as major disruptors of market functioning in pastoralist and agro-pastoralist systems, with direct consequences for food security and nutrition outcomes. Conflict frequently restricted livestock mobility and trader access, leading to market fragmentation, depressed livestock prices, and reduced household purchasing power (80, 81). Studies from conflict-affected regions documented sharp declines in milk sales and food availability, accompanied by rapid increases in food prices and reliance on negative coping strategies (82).

Drought further compounded these impacts by reducing livestock body condition and market value, while simultaneously increasing household dependence on livestock sales to finance food purchases (40). Disease outbreaks affecting cattle, small ruminants, or camels disrupted both production and trade, often triggering temporary market closures that constrained access to both livestock inputs and food commodities (83). Across contexts, the severity of nutrition impacts depended on households’ pre-shock asset positions, herd diversity, and strength of trader networks, with households possessing diversified herds and stronger trader linkages demonstrating greater capacity to buffer shocks.

Conversely, poorer households faced accelerated asset depletion and nutritional decline (48, 84). The synthesis indicates that market resilience shaped by infrastructure, gender dynamics, and governance is a critical but often overlooked component of nutrition resilience in pastoral food systems.

## 6 Sustainability trade-offs

### 6.1 Overgrazing, herd productivity, and competing nutrition needs

Evidence synthesized across the reviewed studies highlights persistent trade-offs between maintaining herd productivity, preventing rangeland degradation, and meeting short- and long-term human nutrition needs in pastoralist and agro-pastoralist systems. Larger herds were frequently associated with improved household food security and dietary diversity through both direct consumption of animal-source foods and increased income from livestock sales; however, when herd sizes exceeded local rangeland carrying capacity, studies consistently documented declines in pasture quality and livestock productivity (85–87). Ayantunde et al. (85) and Cervigni et al. (87) reported that herd expansion beyond rangeland carrying capacity reduced pasture quality and livestock productivity, thereby constraining long-term nutrition benefits. Overgrazing reduced forage availability and nutritional quality, leading to lower milk yields and poorer livestock body condition, which in turn constrained the nutritional benefits derived from livestock ownership (88). These effects were context-dependent and varied according to rainfall variability, herd management practices, and the presence or absence of effective rangeland governance institutions.

Several longitudinal studies emphasized that nutritional gains from herd expansion were rarely linear or sustainable in the absence of effective rangeland management institutions (54). In highly variable dryland environments, herd accumulation was often used as a household strategy to buffer risk and ensure food access during droughts, yet this strategy increased pressure on already fragile ecosystems and amplified vulnerability during prolonged climatic shocks (43, 89). The synthesis suggests that sustainability trade-offs emerge most strongly where short-term food security strategies are pursued without complementary investments in ecosystem stewardship, ultimately undermining livestock productivity and nutrition outcomes over time.

### 6.2 Climate adaptation strategies: herd diversification and fodder banks

Climate adaptation strategies adopted by pastoralist and agro-pastoralist households were widely reported as central to balancing livestock productivity with environmental sustainability and nutrition security. Herd diversification, particularly the inclusion of small ruminants and camels alongside cattle, emerged as one of the most consistently cited adaptive strategies across arid and semi-arid regions (90, 91). Diversified herds were better able to utilize heterogeneous vegetation resources, maintain milk production during droughts, and provide households with flexible assets that could be sold to purchase food without compromising long-term herd viability (92). Studies demonstrated that diversified herd structures were associated with more stable dietary diversity scores across seasons, particularly in drought-prone environments (93).

Fodder banks and strategic feed supplementation were also reported as growing adaptation strategies, particularly among semi-sedentary agro-pastoral households (94). Where implemented effectively, fodder banks reduced dry-season feed gaps, stabilized milk production, and mitigated distress sales of livestock, thereby supporting both nutrition outcomes and rangeland recovery (95). However, evidence suggested that access to fodder banks was uneven and often constrained by land tenure insecurity, labour demands, and limited institutional support (48). Overall, the literature indicates that climate adaptation strategies can reduce sustainability trade-offs only when they are embedded within supportive governance structures, extension services, and explicitly nutrition-sensitive livestock programming, rather than implemented as isolated technical interventions.

### 6.3 Environmental degradation and long-term food security

Environmental degradation emerged as a critical long-term threat to food and nutrition security in pastoralist and agro-pastoralist systems, with rangeland degradation, bush encroachment, and soil erosion repeatedly linked to declining livestock productivity and increased livelihood vulnerability (96–99). Studies emphasized that degradation not only reduced the availability of grazing resources but also eroded customary institutions governing mobility, reciprocity, and resource sharing, key mechanisms through which pastoral systems historically maintained resilience (99). As environmental conditions deteriorated, households reported greater reliance on purchased foods and humanitarian assistance, often accompanied by declining dietary diversity and increased prevalence of chronic undernutrition (30). These trends reflect cumulative pressures on both ecological and social systems rather than the effects of single stressors in isolation.

The synthesis further indicates that environmental degradation interacts with demographic pressure, sedentarization, and climate change to constrain future nutrition pathways (6). In the absence of coordinated rangeland restoration, climate-adapted livestock management, and nutrition-sensitive development policies, current coping strategies risk becoming maladaptive, exacerbating food insecurity rather than alleviating it (12, 42). Collectively, the evidence underscores that long-term food and nutrition security in African drylands is inseparable from environmental sustainability and that resolving livestock-nutrition trade-offs requires integrated approaches that jointly address ecosystem health, livelihood resilience, and human nutrition outcomes within a One Health framework.

### 6.4 Sub-regional variability in nutrition pathways

Evidence across the reviewed studies indicates important sub-regional differences in livestock–nutrition relationships across major African dryland systems. In the Sahel, highly variable rainfall patterns, recurrent droughts, and conflict-exacerbated constraints on pastoral mobility intensify seasonal food insecurity and reduce livestock productivity, often leading to greater reliance on market-mediated food access rather than direct livestock consumption pathways (9, 24, 54). In the Horn of Africa, camel- and cattle-based systems show a strong dependence on milk as a primary nutritional pathway, particularly for children, making households highly vulnerable to drought-induced declines in milk yields and associated increases in wasting and dietary inadequacy (19, 41, 46). In contrast, parts of East Africa, where there is relatively stronger market integration and greater transition toward agro-pastoralism, exhibit more diversified food environments with increased access to cereals, legumes, and market foods, although this diversification is often accompanied by dietary shifts toward purchased and processed foods, reflecting early stages of a nutrition transition (14, 75, 78). These sub-regional differences emphasize that livestock-mediated nutrition pathways are not uniform but are strongly shaped by ecological variability, market access, livelihood transitions, and sociopolitical conditions, and therefore must be interpreted within context-specific environmental and institutional frameworks rather than generalized across dryland systems (12, 27, 34).

## 7 Policy and program implications

### 7.1 Gaps in nutrition-sensitive livestock programming

The synthesis of evidence across this systematic review highlights substantial gaps in the design and implementation of nutrition-sensitive livestock policies in pastoralist and agro-pastoralist systems. Although livestock are widely recognized as central to rural livelihoods in African drylands, many livestock development programs continue to prioritize productivity, commercialization, and disease control without explicit consideration of human nutrition outcomes (27, 49, 70). As discussed in Sections 3–5, increases in herd size, market participation, or income do not automatically translate into improved dietary diversity or child nutrition, particularly in the absence of gender-responsive and seasonally adaptive interventions.

Several reviewed studies emphasized that livestock programs rarely incorporate nutrition indicators such as dietary diversity or child growth into monitoring and evaluation frameworks, limiting their ability to assess impacts beyond income and production metrics (100). Moreover, nutrition-sensitive approaches remain unevenly applied across species, with dairy-focused interventions more frequently linked to nutrition outcomes than small-ruminant or camel-based programs, despite the latter being critical for resilience in highly arid environments (48). These gaps point to the need for livestock policies that explicitly articulate nutrition objectives, pathways, and indicators alongside productivity goals, rather than assuming nutrition gains will emerge indirectly from commercialization alone.

### 7.2 Integrating One Health and multisectoral approaches

The findings of this review strongly support the adoption of integrated One Health and multisectoral approaches that explicitly link animal health, human nutrition, and environmental sustainability in pastoralist and agro-pastoralist contexts. Evidence across multiple sections demonstrates that livestock disease outbreaks, environmental degradation, and market disruptions jointly influence food environments and nutrition pathways (20, 27, 49, 70, 101, 102). Programs that address animal health in isolation risk missing downstream effects on milk availability, food access, and child nutrition, particularly during seasonal and climate-induced stress.

Several studies reviewed highlighted successful examples of integrated interventions, including community-based animal health services combined with nutrition education, milk hygiene improvements, and women-focused income interventions, which together produced more robust and equitable nutrition outcomes than single-sector programs (103). These examples illustrate how coordinated action across livestock, health, and nutrition sectors can generate co-benefits that are rarely achieved through siloed interventions. The adoption of One Health frameworks also offers opportunities to address food safety risks associated with raw milk consumption, zoonotic disease transmission, and antimicrobial use, all of which have implications for public health and nutrition in pastoral systems (104). Institutionalizing multisectoral coordination across livestock, health, nutrition, and environmental ministries remains a critical challenge but is essential for sustainable impact.

### 7.3 Implications for national, regional, and continental policy frameworks

At the policy level, the evidence synthesized in this review underscores the need for stronger alignment between livestock development strategies and broader food systems, nutrition, and climate adaptation agendas at national and regional scales. National agricultural and livestock policies often inadequately reflect the specific realities of pastoralist and agro-pastoralist food systems, particularly the importance of mobility, informal markets, and gendered control of resources (105). Integrating nutrition-sensitive livestock objectives into national development plans can directly support progress toward SDG 2.1 and 2.2 (ending hunger and all forms of malnutrition), SDG 3.2 (reducing preventable child mortality), and SDG 13.1 (strengthening climate resilience) (106, 113).

At the continental level, frameworks advanced by institutions such as the African Union and regional economic communities increasingly recognize the value of pastoralism for food security and resilience, yet implementation remains uneven (107). The findings of this review suggest that future policy efforts should prioritize enabling livestock mobility where ecological conditions demand it, strengthening livestock markets to function during climatic and conflict-related shocks, supporting climate-adaptive herd diversification strategies, and safeguarding women’s control over livestock-derived food and income (108, 109). These priorities are grounded directly in the empirical patterns synthesized in this review rather than in sector-external evidence streams. Embedding these priorities within nutrition strategies, climate adaptation plans, and social protection programs would enhance the contribution of pastoral and agro-pastoral systems to long-term food and nutrition security across African drylands (110, 111).

## 8 Limitations of the review

Despite adherence to PRISMA 2020 guidelines, this systematic review has several limitations that should be considered when interpreting the findings. First, the evidence base is dominated by cross-sectional and observational study designs, limiting the ability to draw causal inferences regarding the relationships between livestock ownership, climate variability, and nutrition outcomes. Associations synthesized in this review therefore reflect reported correlations rather than definitive causal pathways. Second, substantial heterogeneity existed across included studies in terms of study design, nutrition indicators, herd size metrics, livestock species composition, and ecological contexts. Herd size was variously measured using absolute animal counts, Tropical Livestock Units, or locally defined wealth categories, which limited direct comparability and precluded quantitative synthesis or meta-analysis.

Third, screening, eligibility assessment, and data extraction were conducted by a single reviewer, which may increase the risk of selection bias or oversight despite iterative cross-checking against predefined criteria. While this approach is common in interdisciplinary reviews with broad scopes, it nonetheless represents a methodological constraint. Fourth, publication and language bias cannot be excluded, as the review was limited to peer-reviewed literature published in English and French. Relevant studies published in other languages or in grey literature may therefore be under-represented. Finally, the review did not formally assess risk of bias at the individual study level, given the heterogeneity of methods and outcomes and the qualitative nature of the synthesis. Although study quality was considered narratively, residual bias within individual studies may influence the aggregated evidence. These limitations highlight the need for more longitudinal, standardized, and methodologically harmonized research on livestock–nutrition–climate interactions in pastoralist and agro-pastoralist systems.

## 9 Conclusion and future research directions

This systematic review synthesizes evidence from pastoralist and agro-pastoralist systems across Sub-Saharan Africa to examine how livestock holdings, mobility, food environments, markets, gender relations, and environmental dynamics interact to shape human nutrition outcomes in arid and semi-arid lands. The reviewed literature demonstrates that livestock play a significant role in household nutrition through intertwined biological and economic pathways, including direct consumption of animal-source foods, income generation for food purchases, and buffering against seasonal and climate-related shocks. However, these benefits are highly context-dependent and mediated by factors such as herd composition, market access, women’s control over livestock income, mobility, and environmental sustainability.

Despite growing interdisciplinary attention to livestock–nutrition linkages, the evidence base reveals several persistent gaps. First, there is a notable lack of longitudinal studies capable of capturing how livestock ownership, environmental conditions, and nutrition outcomes co-evolve across seasons, shocks, and life-course stages. Most existing studies rely on cross-sectional designs that limit causal inference and obscure long-term trade-offs between livelihoods, ecosystem health, and nutrition. Second, urbanizing and peri-urban pastoralist populations remain under-represented in the literature, despite rapid sedentarization, market integration, and dietary transitions occurring across African drylands. These populations face distinct nutritional risks, including both undernutrition and emerging diet-related non-communicable disease burdens, that differ from those of highly mobile pastoralists. Third, the nutrition and livelihood trajectories of youth in pastoralist and agro-pastoralist systems are poorly documented, even though demographic shifts and changing aspirations are reshaping labour dynamics, livestock management practices, and food environments.

Addressing these gaps will require the development of integrated nutrition–livestock surveillance systems that link routinely collected animal health and production data with human nutrition, food security, and environmental indicators. Such systems would enable real-time monitoring of seasonal and shock-related impacts, support early warning and risk management, and inform the design of nutrition-sensitive livestock and climate-adaptation policies. Future research should prioritize longitudinal, mixed-methods, and systems-based approaches capable of capturing dynamic interactions among climate variability, livestock systems, and nutrition outcomes over time. Strengthening collaboration across livestock, health, nutrition, and environmental sectors using One Health and food-systems frameworks will be critical for sustaining pastoralist and agro-pastoralist livelihoods while improving human nutrition in the face of accelerating climate and socio-economic change.

## Data Availability

The original contributions presented in the study are included in the article/Supplementary material, further inquiries can be directed to the corresponding author.
