# Supplementary material for: Climate variability, pastoral and agro-pastoral livestock systems, and human nutrition in African drylands: a PRISMA-based systematic review of evidence, pathways, and policy implications
Source: Front Nutr. 2026 Jun 16;13:1853901. doi: 10.3389/fnut.2026.1853901 (PMC13314415; doi:10.3389/fnut.2026.1853901)
Supplement: Supplementary file 1 [file Table_1.DOCX]

**Supplementary Tables**

**Table S1: PICOS Framework and Search Scope for the Review**

| **Component** | **Definition Used in This Review** |
| --- | --- |
| Population (P) | Pastoralist and agro‑pastoralist households in Sub‑Saharan Africa |
| Exposure / Intervention (I) | Livestock ownership, herd size/composition, mobility, climate variability, market participation |
| Comparator (C) | Households with lower livestock holdings, restricted mobility, or differing livelihood systems (where applicable) |
| Outcomes (O) | Dietary diversity, ASF intake, child stunting/wasting, micronutrient status, food security |
| Study designs (S) | Quantitative, qualitative, mixed‑methods, cross‑sectional, longitudinal |

**Table S2: Characteristics of Studies Included in the Systematic Review (n = 60)**

| **No.** | **Author(s), Year** | **Country / Region** | **Livelihood System** | **Study Design** | **Sample / Data Source** | **Livestock Exposure** | **Nutrition Outcomes** | **Key Findings Relevant to Review** |
| --- | --- | --- | --- | --- | --- | --- | --- | --- |
| 1 | Randolph et al. [8] | Sub‑Saharan Africa | Pastoral & Agro‑pastoral | Narrative review | Secondary synthesis | Herd size, ASF | Diet diversity, child nutrition | Livestock improve nutrition via ASF and income |
| 2 | Sadler et al., [19] | Ethiopia (Somali Region) | Pastoral | Cross‑sectional + qualitative | HH surveys | Milk intake | Child wasting | Milk central to child diets |
| 3 | Iannotti & Lesorogol, [40] | Kenya (Samburu) | Pastoral | Cross‑sectional | HH surveys | Milk consumption | Child anthropometry | Milk associated with higher HAZ |
| 4 | Nicholson et al. [62] | Kenya (Coastal) | Agro‑pastoral | Quasi‑experimental | HH panel data | Dairy ownership | Food security | Dairy cows raise dietary diversity |
| 5 | Little et al. [25] | East Africa | Pastoral | Mixed methods | Longitudinal HH data | Livestock assets | Food security | Livestock buffer climate shocks |
| 6 | McPeak et al., [10] | Kenya | Pastoral | Panel study | HH panel | Herd dynamics | Food security | Distress sales erode resilience |
| 7 | Thiede & Strube, [111] | SSA | Pastoral & Agro‑pastoral | Quantitative | DHS‑linked data | Climate variability | Child nutrition | Climate variability affects nutrition |
| 8 | Stites & Mitchard, [37] | Uganda (Karamoja) | Pastoral | Mixed methods | HH surveys | Milk access | Diet diversity | Milk markets support diets |
| 9 | Turner et al. [53] | Sahel (West Africa) | Pastoral | Cross‑sectional | HH surveys | Mobility | Food access | Mobility sustains diets |
| 10 | Ayantunde et al. [87] | West Africa | Agro‑pastoral | Comparative | Rangeland & HH data | Herd size | Food security | Overgrazing reduces gains |
| 11 | Krätli et al., [12] | SSA Drylands | Pastoral | Conceptual review | Secondary | Mobility | Nutrition resilience | Mobility supports diets |
| 12 | Njuki et al. [26] | East Africa | Agro‑pastoral | Cross‑sectional | HH surveys | Women’s milk income | Diet diversity | Women’s control improves diets |
| 13 | Hoddinott et al., [70] | Ethiopia | Agro‑pastoral | Cross‑sectional | HH surveys | Market access | Diet diversity | Markets shape diet quality |
| 14 | Fratkin, [63] | Kenya | Pastoral | Mixed methods | Ethnographic | Sedentarization | Diet quality | Settlement alters diets |
| 15 | Catley et al. [47] | Horn of Africa | Pastoral | Case studies | Program data | Vet services | Food security | Integrated programs help nutrition |
| 16 | Lesorogol, [65] | Kenya | Pastoral | Longitudinal | HH panel | Land tenure | Food access | Privatization affects nutrition |
| 17 | Maxwell et al. [45] | Somalia | Pastoral | Mixed methods | Crisis analysis | Livestock loss | Acute malnutrition | Drought worsens nutrition |
| 18 | Grace et al. [38] | SSA | Pastoral | Review | Secondary | ASF intake | Micronutrients | ASF critical in 1 000 days |
| 19 | Turner et al. [39] | West Africa | Agro‑pastoral | Mixed methods | Surveys | Resource conflict | Food security | Conflict disrupts nutrition |
| 20 | Homewood et al., [44] | East Africa | Pastoral | Comparative | HH surveys | Conservation impacts | Diet diversity | Conservation alters livelihoods |
| 21 | Little et al. [46] | Ethiopia | Agro‑pastoral | Longitudinal | Panel data | Drought exposure | Child nutrition | Drought worsens outcomes |
| 22 | Abaynew et al. [61] | Ethiopia | Pastoral & Agro‑pastoral | Cross‑sectional | HH surveys | Livestock assets | Food security | System differences matter |
| 23 | Powell et al. [52] | Tanzania | Agro‑pastoral | Ethnographic | Field surveys | Wild foods | Diet diversity | Wild foods supplement diets |
| 24 | Aklilu & Catley, [54] | Horn of Africa | Pastoral | Policy analysis | Trade data | Livestock export | Income & diets | Markets unevenly benefit poor |
| 25 | McDermott & Grace, [20] | SSA | Mixed | Review | Secondary | Animal health | Nutrition | Health–nutrition links |
| 26 | Fanzo & Davis, [21] | SSA | Mixed | Review | Secondary | Food systems | Diet quality | Systems lens needed |
| 27 | FAO et al. [4] | SSA | Pastoral | Global assessment | National data | ASF access | Undernutrition | ASF gaps persist |
| 28 | Black et al., [31] | Global | Mixed | Meta‑analysis | Global datasets | Diet adequacy | Child mortality | Undernutrition major risk |
| 29 | Wang et al., [110] | Africa | Mixed | Quantitative | Climate‑health data | Climate change | Nutrition risks | Climate alters food–health nexus |
| 30 | Krätli, [67] | Drylands | Pastoral | Review | Secondary | Variability | Resilience | Variability is asset |
| 31 | Rufino et al., [14] | East Africa | Agro‑pastoral | Modeling | HH surveys | Transition pathways | Food security | Mixed systems alter nutrition |
| 32 | Thornton et al., [92] | SSA | Mixed | Review | Secondary | Climate impacts | Food security | Climate affects livestock |
| 33 | Herrero et al., [90] | SSA | Agro‑pastoral | Review | Secondary | Mixed systems | Food security | Mixed systems important |
| 34 | Lybbert et al., [42] | Ethiopia | Pastoral | Longitudinal | Wealth panel | Herd dynamics | Food security | Poverty traps |
| 35 | Maxwell et al. [84] | Somalia | Pastoral | Case study | Crisis analysis | Conflict shock | Nutrition crisis | Conflict worsens hunger |
| 36 | Hesse & Catley, [34] | Africa | Pastoral | Policy report | Secondary | Mobility rights | Food security | Policy affects diets |
| 37 | Galvin, [16] | Africa | Pastoral | Review | Secondary | Transition | Nutrition | Change reshapes food systems |
| 38 | UNDP, [113] | Drylands | Pastoral | Policy analysis | Country data | Adaptation options | Food security | Adaptation supports nutrition |
| 39 | Stringer et al., [112] | Drylands | Pastoral | Review | Secondary | Climate pathways | Nutrition resilience | Resilient development |
| 40 | FAO, [3] | Global | Mixed | Review | Case studies | Feed strategies | Milk production | Feed buffers shocks |
| 41 | Reynolds et al. [98] | Drylands | Pastoral | Review | Secondary | Desertification | Food security | Degradation harms nutrition |
| 42 | Njuki et al. [105] | East Africa | Agro‑pastoral | Review | Case studies | Women’s assets | Diet quality | Gender critical |
| 43 | Quisumbing et al., [71] | Africa | Agro‑pastoral | Review | Project data | Asset control | Child nutrition | Assets improve diets |
| 44 | Ruel & Alderman, [72] | SSA | Mixed | Review | Secondary | Nutrition‑sensitive ag. | Child nutrition | Agriculture–nutrition links |
| 45 | Malapit & Quisumbing, [73] | Ghana | Agro‑pastoral | Quantitative | HH surveys | Women empowerment | Nutrition | Empowerment improves diets |
| 46 | Popkin et al., [80] | Global | Mixed | Review | Global datasets | Diet transition | Double burden | Shifting risks |
| 47 | Seid, [81] | Ethiopia | Pastoral | Case study | HH surveys | Flood risk | Food security | Floods disrupt diets |
| 48 | Maystadt et al., [83] | Sudan | Pastoral | Econometric | Climate datasets | Heat shocks | Food insecurity | Climate–conflict link |
| 49 | Lind et al. [82] | Kenya | Pastoral | Qualitative | Field interviews | Insecurity | Food access | Violence disrupts markets |
| 50 | Haller et al. [64] | Africa | Pastoral | Review | Institutional analysis | Commons change | Diet quality | Institutions matter |
| 51 | Holden & Ghebru, [95] | Global | Agro‑pastoral | Review | Secondary | Land tenure | Food security | Tenure affects diets |
| 52 | Sibanda et al. [96] | Zimbabwe | Agro‑pastoral | Mixed methods | HH surveys | Land use change | Food access | Change affects nutrition |
| 53 | FAO, [97] | Global | Mixed | Review | Program data | Climate‑livestock | Nutrition | Livestock adaptation helps |
| 54 | Scoones & Nori, [107] | Africa | Pastoral | Conceptual review | Case synthesis | Uncertainty | Food security | Living with uncertainty |
| 55 | Dossouhoui et al., [18] | West Africa | Pastoral | Mixed methods | Field surveys | Sedentarization | Food systems | Sedentarization is context‑specific |
| 56 | Thorsen et al., 2021 | Sahel | Pastoral | Qualitative | Interviews | Mobility constraints | Diet quality | Policy affects nutrition |
| 57 | Omosa et al., [13] | Africa | Pastoral | Scoping review | Secondary | Food environments | Nutrition outcomes | Evidence remains fragmented |
| 58 | Krätli & Schareika, [50] | SSA | Pastoral | Conceptual | Secondary | Mobility | Food security | Mobility rational |
| 59 | Cervigni & Morris, [89] | Africa | Drylands | Policy synthesis | Secondary | Resilience strategies | Food security | Integrated approaches needed |
| 60 | Scoones, [108] | Africa | Dryland systems | Review | Secondary | Social‑ecological systems | Nutrition resilience | SES framing useful |
